# Systemic Sympathoexcitation Was Associated with Paraventricular Hypothalamic Phosphorylation of Synaptic CaMKIIα and MAPK/ErK

**DOI:** 10.3389/fnins.2017.00447

**Published:** 2017-08-03

**Authors:** Olalekan M. Ogundele, Fernando A. Rosa, Rohan Dharmakumar, Charles C. Lee, Joseph Francis

**Affiliations:** ^1^Department of Comparative Biomedical Sciences, Louisiana State University School of Veterinary Medicine Baton Rouge, LA, United States; ^2^Departamento de Clínica, Cirurgia e Reprodução Animal, Faculdade de Medicina Veterinária, Universidade Estadual Paulista Araçatuba, Brazil; ^3^Department of Biomedical Sciences, Cedars-Sinai Medical Center Biomedical Imaging Research Institute Los Angeles, CA, United States

**Keywords:** β_2_R, MAPK/ErK, CaMKIIα, TLR4, VGLUT2, GABA, sympathoexcitation

## Abstract

Systemic administration of adrenergic agonist (Isoproterenol; ISOP) is known to facilitate cardiovascular changes associated with heart failure through an upregulation of cardiac toll-like receptor 4 (TLR4). Furthermore, previous studies have shown that cardiac tissue-specific deletion of TLR4 protects the heart against such damage. Since the autonomic regulation of systemic cardiovascular function originates from pre-autonomic sympathetic centers in the brain, it is unclear how a systemically driven sympathetic change may affect the pre-autonomic paraventricular hypothalamic nuclei (PVN) TLR4 expression. Here, we examined how change in PVN TLR4 was associated with alterations in the neurochemical cytoarchitecture of the PVN in systemic adrenergic activation. After 48 h of intraperitoneal 150 mg/kg ISOP treatment, there was a change in PVN CaMKIIα and MAPK/ErK expression, and an increase in TLR4 in expression. This was seen as an increase in p-MAPK/ErK, and a decrease in synaptic CaMKIIα expression in the PVN (*p* < 0.01) of ISOP treated mice. Furthermore, there was an upregulation of vesicular glutamate transporter (VGLUT 2; *p* < 0.01) and a decreased expression of GABA in the PVN of Isoproterenol (ISOP) treated WT mice (*p* < 0.01). However, after a PVN-specific knockdown of TLR4, the effect of systemic administration of ISOP was attenuated, as indicated by a decrease in p-MAPK/ErK (*p* < 0.01) and upregulation of CaMKIIα (*p* < 0.05). Additionally, loss of inhibitory function was averted while VGLUT2 expression decreased when compared with the ISOP treated wild type mice and the control. Taken together, the outcome of this study showed that systemic adrenergic activation may alter the expression, and phosphorylation of preautonomic MAPK/ErK and CaMKIIα downstream of TLR4. As such, by outlining the roles of these kinases in synaptic function, we have identified the significance of neural TLR4 in the progression, and attenuation of synaptic changes in the pre-autonomic sympathetic centers.

## Introduction

Paraventricular hypothalamic adrenergic neurotransmission forms part of the pre-autonomic control of systemic cardiovascular function (Xu et al., 2012; Lee et al., 2013; Patel et al., 2016). Furthermore, abnormalities in the expression of β-adrenergic receptors (β*AR)* or its hyperactivation in cardiac tissue have been described in rodent models of heart failure (Madamanchi, 2007; Giudice et al., 2016; Yamazaki et al., 2016).

The neural control of systemic cardiovascular function is mediated through multiple feedback loops involving the preautonomic sympathetic centers of the brain and peripheral mechanoreceptor systems. As such, an increase in the activity of the PVN, or brain stem projections to the intermediolateral gray column may increase heart rate. Evidently, an increase in sympathetic activation promotes the release of norepinephrine, which activates cardiac β-adrenergic receptors.

*βARs* are G-protein coupled receptors expressed on the surface of neurons, cardiac cells, hepatocytes, endothelial cells and adrenal glands (Ichikawa et al., 2016; Romberger et al., 2016). In the presence of a βAR agonist, such as isoproterenol (ISOP), G-protein is released leading to the activation of *PIP*_3_ which initiate cascades of signaling events involving the phosphorylation of downstream kinases (Dawaliby et al., 2016; Ranjan et al., 2016). In support of this proposition, the transcription of *Ras/Raf/MAPK* genes increases after ISOP treatment and is associated with TLR4 signaling (Kim et al., 2006; Lu et al., 2014; Theccanat et al., 2016). In addition, the expression of TLR4 in cardiac tissue may determine the efficacy of βAR agonists as (such as ISOP) agents to induce heart failure. As such, a cardiac tissue-specific deletion of TLR4 prevents cardiac tissue damage after ISOP treatment (Kim et al., 2006).

The relative abundance of β*ARs* on various cell types in the nervous and vascular system makes it (that is β*ARs*) a suitable target for disease models and therapeutic targeting (Badshah et al., 2016; Ranjan et al., 2016). Furthermore, activation of *Ras/Raf*-downstream of β*ARs*-suggests the involvement of pro-inflammatory receptors and associated pathways in systemic cardiovascular changes (Cho et al., 2016). Although previous studies have described changes in systemic inflammatory response in heart failure models, how a systemic driven change may affect the neurochemical architecture of the preautonomic centers is yet to be elucidated.

Previous studies have shown that βAR signaling promotes inflammation and sympathoexcitation simultaneously (Mo et al., 2014; Morioka et al., 2014). Through its effect on *TLR4* and NMDAR, βAR affects nNOS, and GABA neurotransmission in the brain; further increasing excitatory glutamatergic potentials (Sharma et al., 2011; Lethbridge et al., 2012; Di Mauro et al., 2013). Similarly, TLR4-mediated MAPK/ErK activation leads to inflammation via phosphorylation of calcium-calmodulin dependent kinase II alpha (CaMKIIα) (Mizukami et al., 2015; Yu et al., 2016). Thus, we hypothesize that attenuating MAPK/ErK inflammatory signaling events, in the PVN neurons, may reduce the severity of sympathoexcitation by reducing the phosphorylation of CaMKIIα.

Despite the available evidence on the role of TLR4 in βARA-mediated tissue damage, the significance of neural (PVN) TLR4 during systemic βARA remains unknown. In this study, we sought to evaluate the effect of systemically driven βAR activation on the neurochemical cytoarchitecture of the PVN. Specifically, the study highlights the significance of PVN TLR4 in altering synaptic kinases, excitatory, and inhibitory neurotransmitter balance after systemic βAR activation.

## Methods

### WT and transgenic mice strains

Adult C57BL/6 (WT; *n* = *20*) and transgenic C57BL/TLR4^*loxp*/*loxp*^ (*n* = 20) were procured from the Jackson Laboratory, United States. C57BL (VGAT)-Venus mice (*n* = 10), previously characterized by Wang et al. (2009), were obtained from Dr. Janice Nagle at Wesleyan University. All animals used for this experiment weighed between 22 and 25 g. Animals were kept under standard laboratory condition and handled using NIH guidelines for animal care and use in research. All protocols used were reviewed and approved by the Institutional Animal Care and Use Committee of the Louisiana State University.

### Anatomical tract tracing

The projections from the *PVN* were identified using fluorescent retrograde anatomical tract tracing and immunohistochemistry. Flame pulled glass pipettes were back-filled with mineral oil and mounted on a Nanoject (Drummond Instruments). The tip of the pipette was trimmed with forceps to increase the width to 200 μm and reduce resistance to the injected bolus. Rhodamine B + Amine [10,000 MW] (*Life Technologies;* Molecular Probe) was diluted (10%) in saline and drawn into tip of the glass pipette. The coordinate of the Nucleus of solitary tract (NTS) was determined using a stereotaxic apparatus (NTS: Anteroposterior −6.96 mm, Mediolateral +1.35 mm; Franklin and Paxinos, 1997). A dental drill was used to remove the bone on the marked AP/ML intersection until the dura became visible. The exposed brain area was kept moist with oxygenated artificial cerebrospinal fluid [aCSF; in mM 125 NaCl, 25 NaHCO_3_, 3 KCl, 1.25 NaH_2_PO_4_, 1 MgCl_2_, 2 CaCl_2_, and 25 Glucose] throughout the duration of the experiment. The pipette (Nanoject) was lowered gradually into the NTS (Dorsoventral: +5.5 mm) following which two boli (5 nL each) were injected at 120 s interval. After a resting period of 3 min, the pipette was lowered further (DV: +6.0 mm) to label the SolL/SolV area. After a 7-day survival period, animals were euthanized following which the whole brain was removed and kept in ice-cold oxygenated aCSF. Vibrotome sliced coronal slices (500 μm thick) exposing the *PVN* (Bregma −0.58 mm to −1.22 mm) or ***NTS*** were fixed in 4% 10 mM phosphate buffered paraformaldehyde (4% PB PFA). In subsequent processing, 60 μm thick cryostat sections were obtained from the fixed slices and labeled further in immunofluorescence methods.

### Adenoviral gene expression and tissue specific PVN TLR4 knockdown

AAV-CMV-eGFP and AAV-CMV-*Cre*-eGFP were procured from the University of Iowa Vector Core and stored at −80°C. TLR4^*loxp*/*loxp*^ mice were anesthetized using Ketamine/Xylazine (100mg/Kg:10mg/Kg body weight). The PVN was targeted stereotaxically for injections at coordinates relative to the bregma [AP −0.85, ML + 1.5 mm, DV +5.5 mm] (Franklin and Paxinos, 1997). Control (AAV-CMV-eGFP; *n* = 10 TLR4^*loxp*/*loxp*^ mice) or *Cre*-expressing AAV (AAV-CMV-*Cre*-eGFP; *n* = 10 TLR4^*loxp*/*loxp*^ mice) was injected into the PVN (500 nL) using a low resistance flame pulled glass pipette (200 μm wide; Lee et al., 2017). The pipette was filled with mineral oil and mounted on a Nanoject (Drummond Instruments, USA). After exposing the dura at the designated coordinates, adenoviral cocktail was aspirated into the tip of the pipette and checked for flow before being lowered into the neural tissue. The injections were done in multiple boli of *23 nL each* at 60 s interval. After the last injection, the pipette stayed in position for 180 s before it was gradually withdrawn. AAV gene expression (eGFP reporter) was verified 3 weeks after the injection in wet mount brain slices; using an Olympus BX 51 Fluorescence microscope.

### βAR activation in WT and transgenic mice

Isoproterenol (Sigma, USA) was diluted in normal saline and administered in two separate intraperitoneal injections of 150 mg/kg (per injection) per animal (24 h′ interval; Gupta et al., 2013). ISOP treatment was done in the following groups; WT/ISOP (*n* = 10), TLR4^*loxp*/*loxp*^/ISOP (*n* = 10) and TLR4^*Cre*/−^/ISOP (*n* = 10). The animals were euthanized 24 h after the last injection to obtain brain (PVN) sections for immunofluorescence. A separate group of *n* = 10 WT mice were treated with normal saline (i.p).

#### Echocardiography

Cardiac function was measured using Toshiba Aplio echocardiographic machine as described previously (Dange et al., 2014).

### Whole-cell patch-clamping electrophysiology

To assess physiological effects in the brains of some animals, after 48 h of ISOP acute treatment, animals were decapitated following which the whole brain was removed and kept in ice-cold oxygenated artificial cerebrospinal fluid (ACSF; in mM 125 NaCl, 25 NaHCO_3_, 3 KCl, 1.25 NaH_2_PO_4_, 1 MgCl_2_, 2 CaCl_2_, and 25 Glucose). Vibratome sectioned coronal brain sections (400 μm thick) containing the *PVN* (Bregma −0.58 to −1.22 mm) were prepared in cold oxygenated artificial cerebrospinal fluid (ACSF) and transferred to oxygenated ACSF at 34°C for recovery (1 h). Subsequently, the brain slices were moved to a perfusion chamber mounted on an Olympus BX 51 phase contrast microscope. To record from neurons using the perforated patch technique, freshly aliquoted 3 mg of Amphotericin B was dissolved in 50 μl of dimethyl sulfonate (DMSO) and stored at −20°C. Subsequently, 8 μl of Amphotericin B solution was added to 2 ml of intracellular pipette solution in mM 135 Potassium gluconate, 7 NaCl, 10 4-(2hydroxy ethyl)-1-piperazineethanesulfonic gluconate, 1-2Na_2_ATP, 0.3 Guanosine Trisphosphate (GTP) and MgCl_2;_ the pH was adjusted with KOH and final Osmolality was set at 290 mOsm; Amphotericin B (0.24 mg/ml)] following which it was kept on ice and stored away from light. Flame pulled glass pipette electrode (with resistance 3–5 MΩ) were prepared on a P-97 pipette puller (Stutter Instruments) and filled with the intracellular solution containing Amphotericin B. Using a micromanipulator, the electrode was visually guided to create a perforated patch (12–15 min) to isolate single-cell potentials (mV) in current clamp mode. Only cells with access resistance of 5–20 MΩ were recorded. Frequency (Hz) of evoked membrane depolarization potential was detected using Multiclamp 700B Amplifier and Digidata 1440A digital acquisition system (Molecular Instruments) after a step current was injected (60pA). Analysis of evoked action potentials was done in ClampFit (Axon Instrument, Sunnyvale, CA).

### Western blotting and protein quantification

For western blot analyses, 15 μl brain tissue lysate containing 15 μg of protein was processed for SDS-PAGE electrophoresis for *n* = 7 samples per group. After subsequent western blotting (wet transfer), Polyvinylidene fluoride membrane (PVDF) was incubated in Tris-buffered saline (with 0.01% Tween 20) (TBST) for 15 min in TBST at room temperature. Subsequently, the membrane was blocked in 3% Bovine serum albumin (prepared in TBST) for 50 min at room temperature. The protein of interest, and control (Homer-1 and GAPDH) were detected using the following primary antibodies; Mouse anti-CaMKIIα (Cell Signaling-#50049), Rabbit anti p-CaMKIIα (Cell Signaling- #12716S), Rabbit anti MAPK/ErK (Cell Signaling- #9102S), Rabbit anti p-MAPK/ErK (4370S), Rabbit anti NMDAR1 (abcam-# ab17345), Rabbit anti GABA_B_R (abcam-#ab166604), Rabbit anti GAPDH (Cell Signaling-#517S), Rabbit anti Homer1 (Cell Signaling-#8231), Mouse anti TLR4 (abcam-# 76B357.1) and Rabbit anti NeuN-Alexa 488 Conjugate (abcam-#ab190195). All primary antibodies were diluted in the blocking solution at 1:1,000. Subsequently, the primary antibodies were detected using HRP-conjugated secondary antibodies (goat anti-rabbit-#65-6120 and goat anti-mouse-#65-6520; Invitrogen; dilution of 1:5,000 or 1:10,000) following which the reaction was developed using a chemiluminescence substrate (Thermo Fisher-#34579).

### Cardiac histology

Cardiac tissue was stained in Hematoxylin and Eosin (*n* = 5 per group) to demonstrate the general morphology of the ventricular wall using the methods of Fischer et al. (2008). Images were acquired in Nanozoomer (Hamatsu, USA). The affected area was determined in using Image J (NIH, USA).

### Immunohistochemistry

WT and transgenic mice (*n* = 10 per group) were deeply anesthetized in isoflurane and perfused transcardially through the left ventricle using 10 mM phosphate buffered saline. Subsequently, 4% phosphate buffered paraformaldehyde (PB-PFA) was introduced for perfusion fixation. The whole brain was harvested through cranial dissection and fixed in 4% PB-PFA overnight, following which it was transferred to 4% PB-PFA containing 30% Sucrose for cryopreservation. Free-floating cryostat sections (40 μm) were collected into 10 mM PBS stored away from light (4°C). Before the commencement of staining, the sections were washed 3 times (5 min each) in 10 mM PBS (pH 7.4). To block non-specific binding, sections were incubated in normal Goat serum (Vector Labs), prepared in 10 mM PBS (with 0.03% Triton-X 100), at room temperature for 1 h. Subsequently, the sections were washed 3 times in PBS and moved to a primary antibody solution for overnight incubation at 4°C. Primary antibodies [Mouse anti-CaMKIIα (1:100; Cell Signaling-#50049), Guinea pig anti-VGLUT 2 (1:250; EMD Millipore-AB2251), Rabbit anti-GABA (1:200; Abcam-ab8891), Rabbit anti-MAPK/ErK1/ErK2 (1:100; Cell Signaling-#9102)] were prepared in 10 mM PBS, 0.03% Triton X-100 and Normal Goat Serum. In further processing, the sections were washed (as described previously) and incubated in secondary antibody solution [Goat anti-Rabbit Alexa-568, Goat anti-Guinea pig Alexa-568, and Goat anti-Mouse Alexa-568 (1:1,000), 10 mM PBS, 0.03% Triton X-100 and Normal Goat Serum] for 1 h at room temperature in a dark enclosure. Labeled sections were washed and mounted on gelatin coated slides with plain anti-fade mounting medium (Vector Labs).

### Confocal microscopy and cell quantification

Imaging of adenoviral (AAV-CMV-eGFP or AAV-CMV-Cre-eGFP) gene reporter expression (*eGFP*) was performed on serial cryostat coronal brain section (400 μm) using an Olympus BX 51 florescence microscope. The distribution of immunolabeled proteins (MAPK/ErK1/ErK2, CaMKIIα, GABA, and VGLUT 2) in the PVN were determined using confocal microscopy (Olympus FluoView 10i). To demonstrate cellular changes in inhibitory neurons (Venus) of the PVN, whole brain confocal scans and montaged higher magnification images were generated for (VGAT)-Venus mice after ISOP treatment. Immunolabeled cells and proteins were counted in confocal PVN images (*n* = 5 per group per protein) using Image J (NIH, USA). Furthermore, the average count per unit area was estimated bilaterally relative to the third ventricle. For each PVN on a section, the count was determined for *n* = 7 areas (per μm^2^) in different field of views. We determined the limits of the PVN using cellular morphology characterization and region-dependent cell density. This was combined with retrograde tracing (as above) to determine the approximate PVN region projecting to the NTS.

### Statistics

The average values for cell counting per unit area and fluorescence yield were compared for WT vs. WT/ISOP, TLR4^*loxP*/*loxP*^/ISOP vs. TLR4^*Cre*/−^/ISOP, and (VGAT)-Venus/ISOP vs. (VGAT)-Venus /Saline (control) mice using Student's *t-*test (GraphPad Prism Version 5). The outcome of statistical analysis was presented in bar chart with error bar representing the mean ± SEM.

## Results

### Retrograde labeling

**Figure 1:**
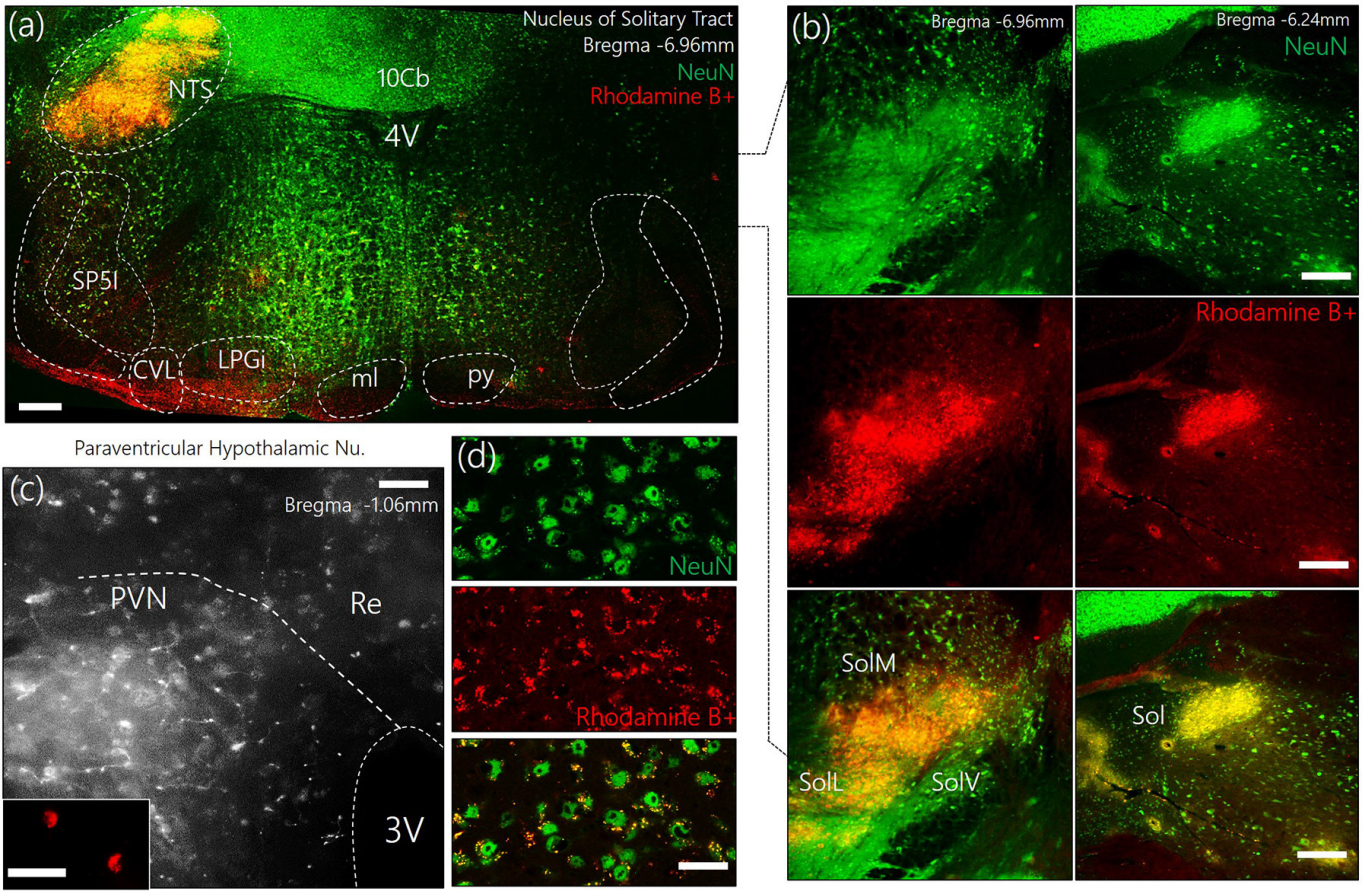
**(a)** Representative confocal images showing the injection site for the retrograde label—Rhodamine B+ Amine (10,000 MW)—in the Nucleus of tractus solitarius. NTS, Nucleus of tractus solitarius; Sol, Solitary tract (M: Medial, L: Lateral, V: Ventral); SP5I, spinal trigeminal nucleus (interploar part); ml, medial lemniscus; py, pyramidal tract; 4V, fourth ventricle; 10cb, lobule of 10th cerebellar vermis; CVL, caudoventrolateral reticular nucleus; LPGi, lateral paragigantocellular nucleus. **(b)** Fluorescence images showing the co-localization of Rhodamine and NeuN (neurons) in the posterior and anterior parts of the Sol (scale bar = 50 μm). **(c)** Confocal images showing the distribution of Rhodamine labelled retrograde vesicles in the PVN after a 7-day survival period (scale bar = 50 μm). (*Inset*) Localization of Rhodamine in the PVN neurons (scale bar = 30 μm). **(d)** Co-localization of retrograde labels and neurons (NeuN) in the PVN (scale bar = 20 μm).

To identify the approximate localization of pre-autonomic sympathetic neurons, we employed retrograde anatomical tracing methods to outline of the PVN after a dye was injected into the NTS (Figures 1a,b). The outcome of this experiments showed that retrogradely labeled cells were relatively abundant in the superiomedial aspect of the PVN. As shown in Figures 1c,d, retrograde vesicles were co-localized with neuronal cell bodies labeled with NeuN.

### Systemic βARA altered synaptic kinases expression in the PVN of WT mice

**Figure 2:**
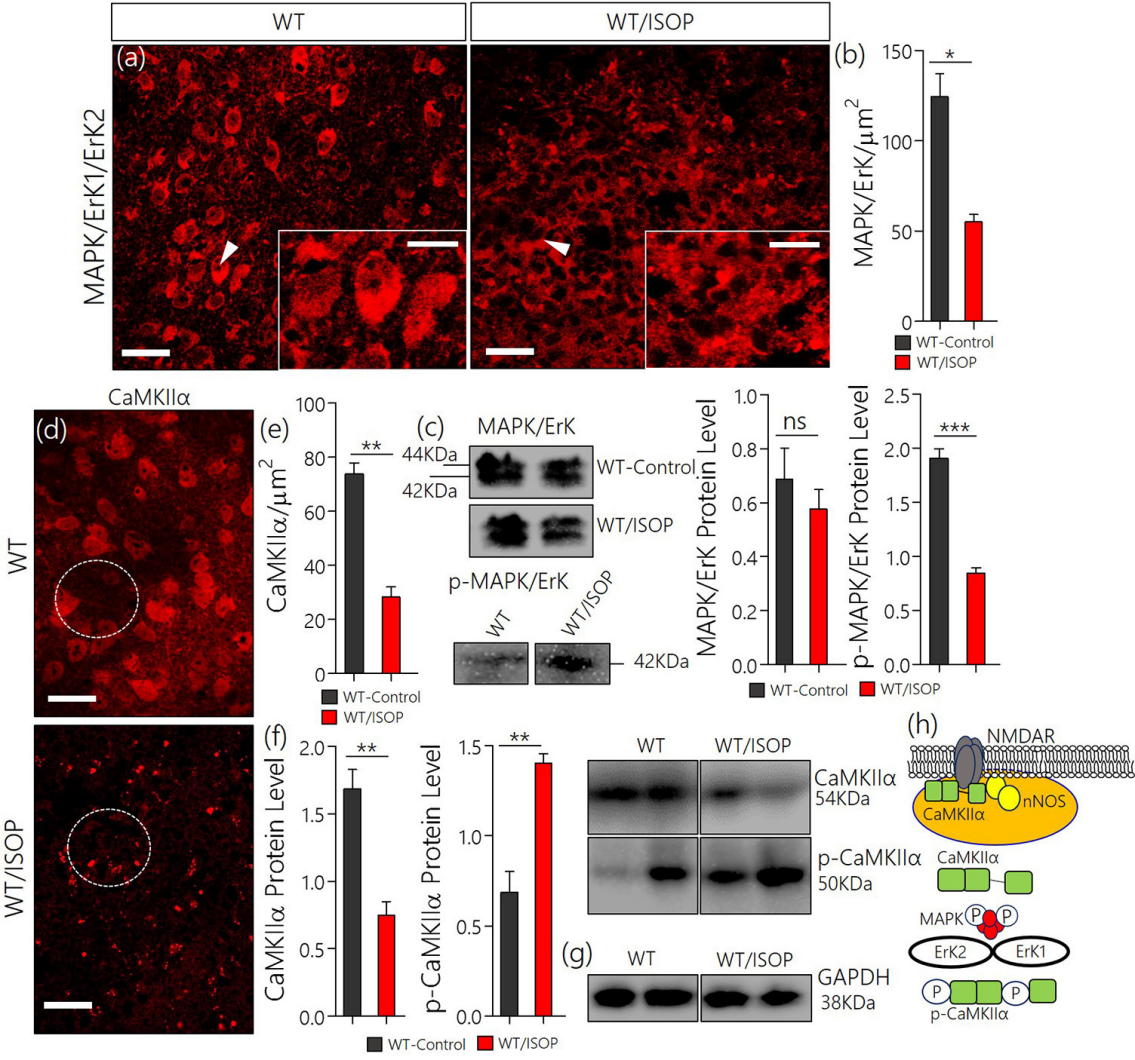
**(a)** MAPK/ErK1/ErK2 expression is altered in the PVN of ISOP treated WT (WT/ISOP) mice when compared with the control (^**^p < 0.01; scale bar = 20 μm, 5 μm). Arrow head depict the relative expression of MAPK/ErK in PVN cells. **(b)** Representative bar chart showing the statistical significance of change in MAPK/ErK expression in the PVN after a systemic βARA. **(c)** Immunoblots showed no significant change in total MAPK/ErK (ns), but an increase in phosphorylated MAPK/ErK (p-MAPK/ErK) in the PVN of ISOP treated WT mice; vs. the total MAPK for the ISOP treated group (^***^p < 0.001). **(d)** Up-regulation of the p-MAPK/ErK1/ErK2 was associated with a decrease in PVN CamKIIa expression after 48 h of ISOP treatment (^*^p < 0.05; scale bar = 20 μm, 5 μm). **(e)** Bar chart depicting the expression level of CaMKIIa in the PVN of control and ISOP treated WT mice. **(f)** Immunoblots showing a decrease in total CaMKIIa for the WT/ISOP group when compared with the control (^**^p < 0.01). As such, there was a significant increase in phosphorylated CaMKIIa vs. the control (^**^p < 0.01). **(g)** Quantitative expression of control proteinGAPDHfor control (WT) and ISOP treated groups. **(h)** Schematic illustration of the possible mechanism through which MAPK and CaMKIIa are regulated by phosphorylation downstream of TLR4. Additionally, we depicted a proposed mechanism by which there interaction may alter synaptic activity of NMDAR and nNOS.

After βARA, in confocal microscopy, we found a significant decrease in total MAPK/ErK expression within the PVN of the WT/ISOP group when compared with the control (Figures 2a,b; *p* < 0.05). However, the expression of phosphorylated MAPK/ErK increased in the WT-ISOP group when compared to the total MAPK expression in quantitative immunoblotting (Figure 2c; *p* < 0.05). Overall, the total MAPK expression reduced significantly for the WT/ISOP group when compared with the control (Figures 2a–c). Alterations in MAPK/ErK and p-MAPK/ErK were accompanied by a significant loss of CaMKIIα in the PVN neurons of ISOP treated mice when compared with the control (Figures 2d,e). Interestingly, in quantitative protein analysis, we recorded a significant decrease in PVN CaMKIIα (*p* < 0.01), and an increase in phosphorylated CaMKIIα (*p* < 0.01) when the WT/ISOP group was compared with the control (Figures 2f,g). This suggests that a change in systemic sympathetic βARA elicited neural changes in PVN by altering the expression of pattern of kinases involved in the regulation of synaptic function within the PVN (Figure 2h). Specifically, this involved a preferential expression of p-MAPK/ErK and downregulation of CaMKIIα through phosphorylation (increased p-MAPK/ErK).

### Systemic βARA altered the PVN glutamate/GABA expression pattern

**Figure 3:**
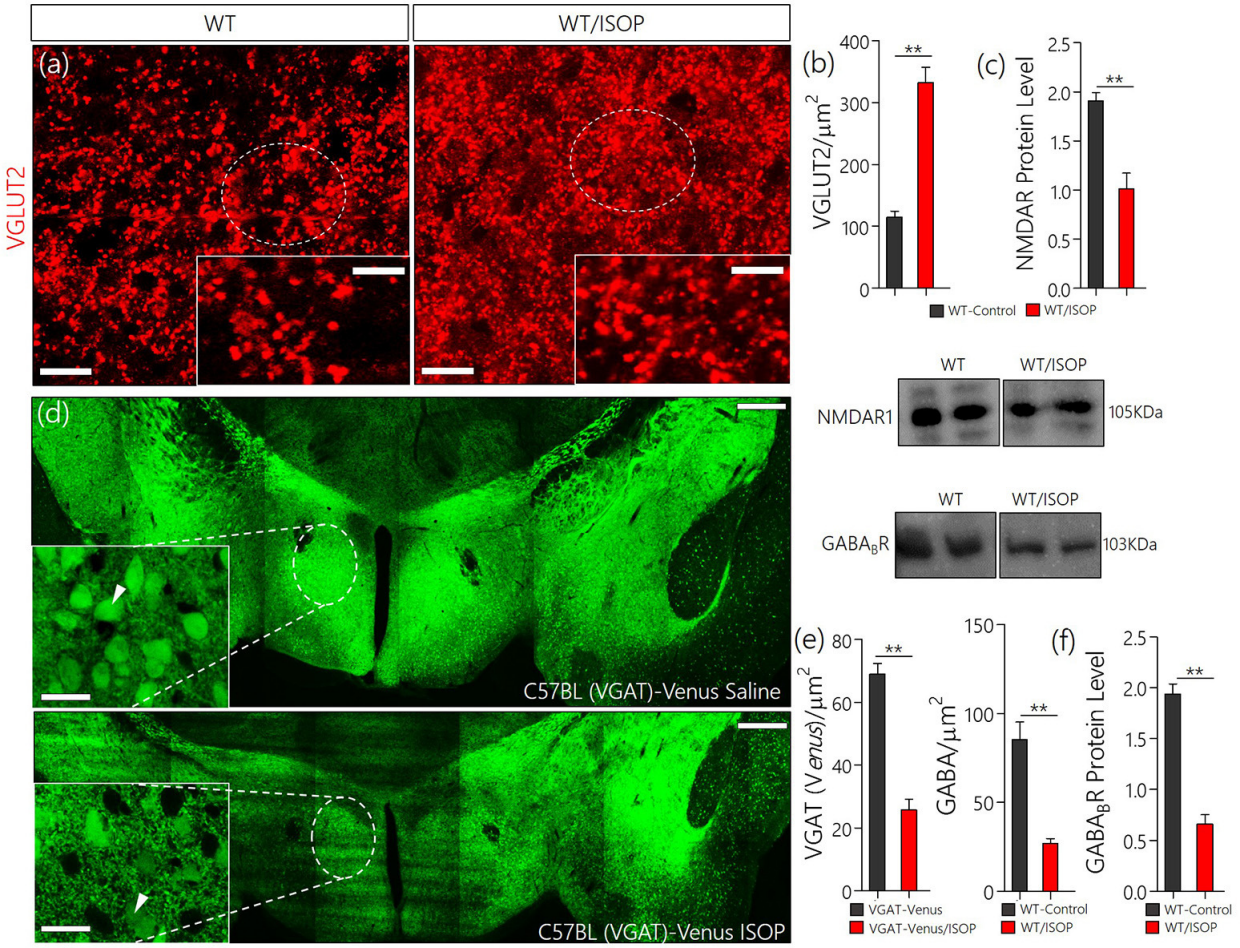
**(a)** Vesicular glutamate transporter expression (VGLUT 2) in the PVN of ISOP treated WT mice vs. the control (^**^p < 0.01; scale bar = 10 μm). **(b)** Bar chart showing an increase in VGLUT2 within the PVN of ISOP treated WT mice. **(c)** Immunoblots showing downregulation of NMDAR in the PVN of WT/ISOP treated mice; vs. the control (^**^p < 0.01). **(d)** Representative confocal image of the PVN for control and ISOP treated transgenic C57BL (VGAT)-Venus mice; expressing Venus in the inhibitory neurons. Subsequent quantification indicates a decrease in the yield of inhibitory neurons in the PVN of ISOP- vs. saline-treated mice (^**^p < 0.01; scale bar = 200 and 10 μm). Arrow heads indicate Venus positive neurons in the PVN. **(e)** Statistical representation of Venus positive cell count in the PVN of control and ISOP treated (VGAT)-Venus mice. **(f)** Quantitative protein analysis for PVN GABA_B_R expression. Like VGAT, there was a significant decrease in GABA_B_R expression in the PVN vs. the control (^**^p < 0.01).

In subsequent experiments we evaluated the significance of changes in MAPK/CaMKIIα on components of the PVN glutamate/GABA system. Here we showed the distribution of transporters (VGLUT2 and VGAT) and associated receptors (NMDAR and GABA_B_R) in the PVN of control and ISOP treated mice (WT/ISOP). In addition to a change in MAPK/ErK/CaMKIIα, ISOP treated mice showed a significant upregulation of VGLUT 2 (Figures 3a,b; *p* < 0.01), but a decrease in NMDAR expression in the PVN when compared with the control (Figure 3c; *p* < 0.01). In a separate experiment, transgenic (VGAT)-Venus mice, which express the *Venus* fluorescent protein (an enhanced form of YFP) in inhibitory GABAergic and glycinergic neurons, were treated with ISOP to determine the effect of βARA on inhibitory neurons in the PVN. A significant decrease in *Venus* fluorescence intensity was observed in the PVN of ISOP treated (VGAT)-Venus mice when compared with control (VGAT)-Venus group (Figures 3d,e). Furthermore, there was a significant loss of GABA_B_R in the PVN (*p* < 0.01; Figure 3f). Based on these outcomes, we deduced that that acute systemic βAR activation promotes pre-autonomic glutamatergic vesicular activity, while suppressing inhibitory neurotransmission.

### Sympathoexcitation in βAR activation

**Figure 4:**
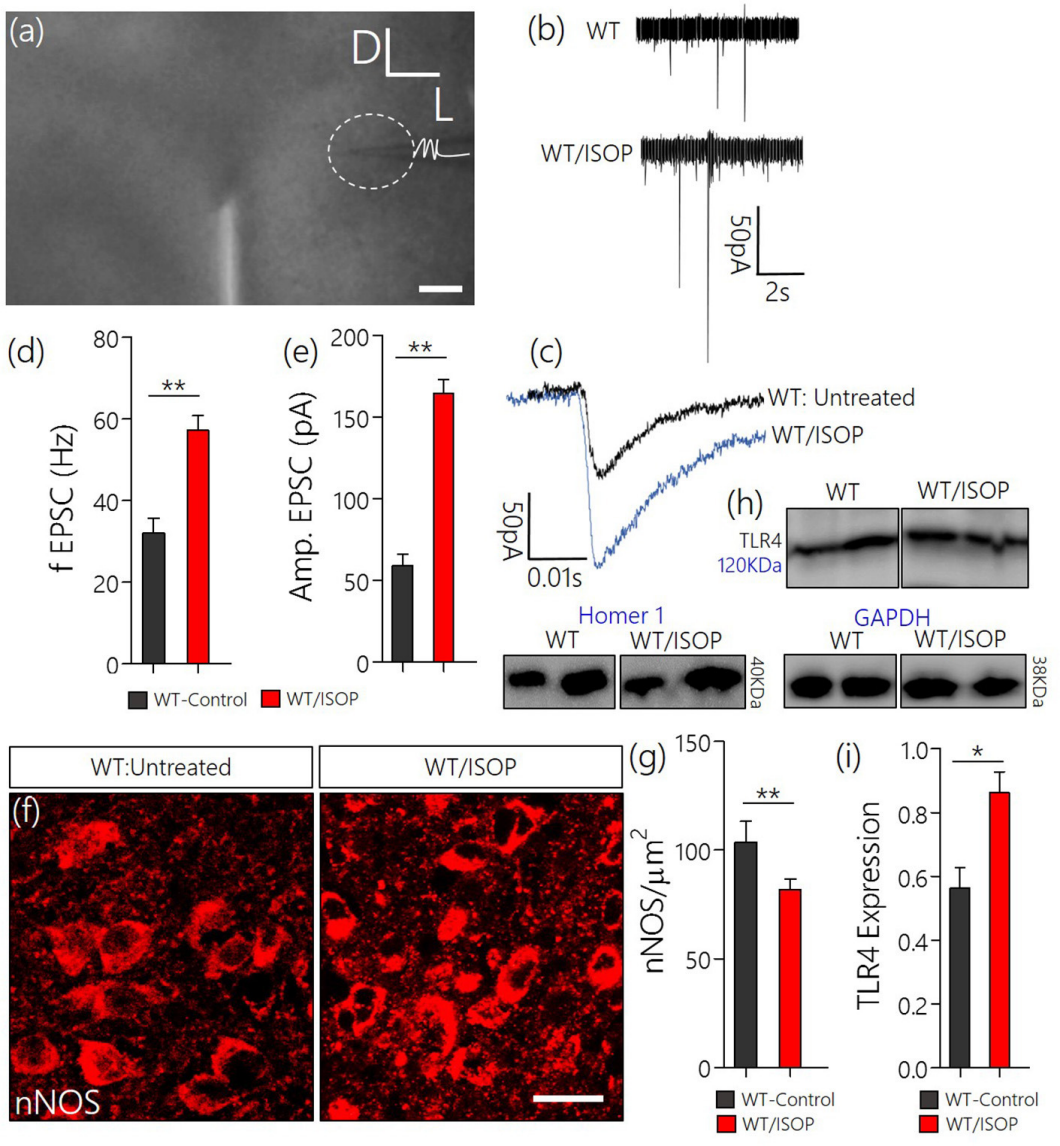
**(a)** Phase contrast image showing the relative placement of patch pipette electrode in acute slice (PVN) physiology (Scale bar = 100 μm). **(b,c)** Spontaneous EPSCs recorded from the PVN of control mice, and mice previously treated with ISOP (in vivo). ISOP treated WT mice showed an increase in the frequency (^**^p < 0.01) and amplitude (^**^p < 0.01) of spontaneous EPSCs when compared with control (untreated WT) PVN neurons. **(d,e)** Bar chart depicting a significant increase in the frequency (^**^p < 0.01) and amplitude (^**^p < 0.01) of EPSCs in the PVN of ISOP treated mice. **(f,g)** Representative confocal images showing a decrease in nNOS in the PVN after a systemic ISOP treatment (Scale bar = 20 μm). This further supports a change in NMDAR function and synaptic stress after an acute systemic adrenergic activation. **(h)** In addition to a decrease in nNOS, TLR expression increased in the PVN of WT/ISOP mice (^*^p < 0.05). **(i)** Bar chart showing the normalized TLR4 expression for control (WT) and WT/ISOP groups.

Since we have shown that a systemic adrenergic activation may cause certain indirect effects on preautonomic function, we verified this observation by determining the profile of spontaneous EPSCs in acute slices prepared from ISOP treated mice (Figure 4a). Using whole-cell perforated patch recordings from the PVN of WT/ISOP group (*n* = 5), we found an increase in spontaneous EPSCs when compared with the control (*p* < 0.01). Additionally, there was an increase in the amplitude of the EPSCs (*p* < 0.01) vs. the control (Figures 4b–e). In support of this outcome, we quantified the expression of nNOS as an indicator of sympathoexcitation that may be related to both NMDAR and GABA_B_R function. Interestingly, we found a decrease in nNOS in the PVN of WT/ISOP groups when compared with the control (*p* < 0.01; Figures 4f,g). Ultimately, these changes were associated with an increase in PVN TLR4 expression (*p* < 0.05; Figures 4h,i).

### Tissue-specific knockdown of PVN TLR4 reduced systemic βAR-mediated MAPK/ErK expression

**Figure 5:**
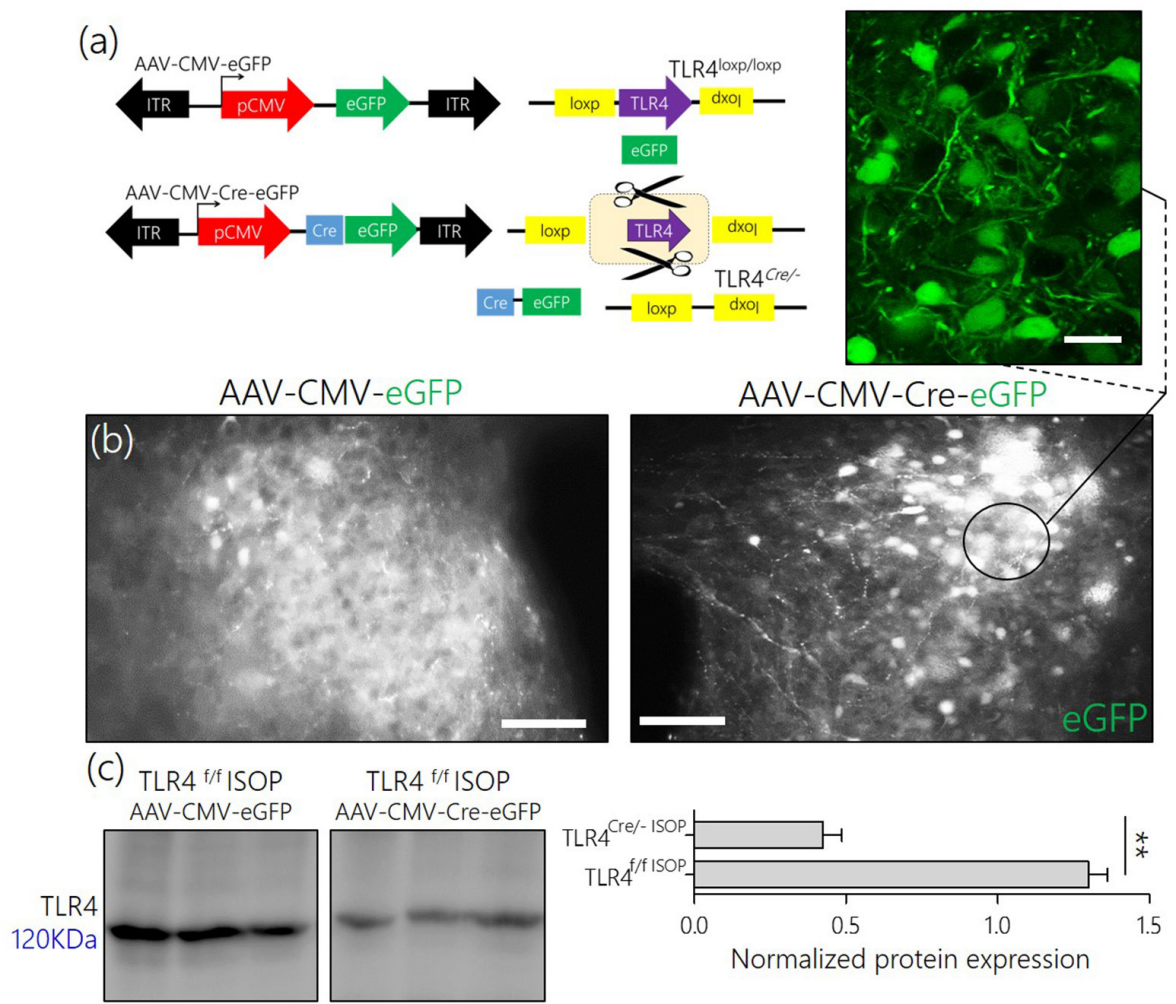
**(a)** AAV-eGFP expression in the PVN TLR4^*F*/*F*[*loxp/loxp*]^ Mice. Schematic representation of control (AAV-CMV-eGFP) and *Cre*-dependent (AAV-CMV-Cre-eGFP) knockout of TLR4 in the PVN of TLR4^*loxp*/*loxp*^ mice. **(b)** AAV-CMV-eGFP and AAV-CMV-Cre-eGFP expression in the PVN; 3 weeks after stereotactic injection. Fluorescence images showing the expression of eGFP reporter in the PVN (scale bar = 20 μm, 10 μm). The control phenotype remained TLR4^*loxp*/*loxp*^ while the PVN neurons expressing AAV-CMV-Cre-eGFP depicts the phenotype TLR4^*Cre*/−^. **(c)** Normalized expression of TLR4 detected through Immunoblotting of PVN lysate. A significant knockdown of TLR4 was recorded in the PVN of TLR4^*Cre*/−^ mice when compared with the TLR4^*loxp*/*loxp*^ PVN.

From these findings, we hypothesized that an alteration of synaptic kinases may be responsible for the overall change in synaptic function associated with a systemic βARA. Since the balance between CaMKIIα and MAPK/ErK expression, and phosphorylation, is involved in inflammatory receptor (TLR4) signaling, we asked whether manipulation of TLR4 in the PVN reduces the threshold of MAPK/CaMKIIα imbalance and PVN sympathoexcitation (Figure 5a).

After stereotactic injection of AAV-cocktail in the PVN, successful AAV gene transfection was confirmed through fluorescence detection of the reporter (eGFP) for AAV-CMV-eGFP and AAV-CMV-Cre-eGFP in the PVN after 21 days (Figure 5b). To confirm the knockdown of TLR4 in the PVN of TLR4^*loxp*/*loxp*^ mice, we estimated TLR4 expression using western blotting analysis. Our results showed a significant reduction in TLR4 expression in untreated TLR4^*Cre*/−^ when compared with the untreated TLR4^*loxp*/*loxp*^ PVN (Figure 5c).

### TLR4*^*Cre*/−^* attenuated MAPK/CaMKIIα imbalance

**Figure 6:**
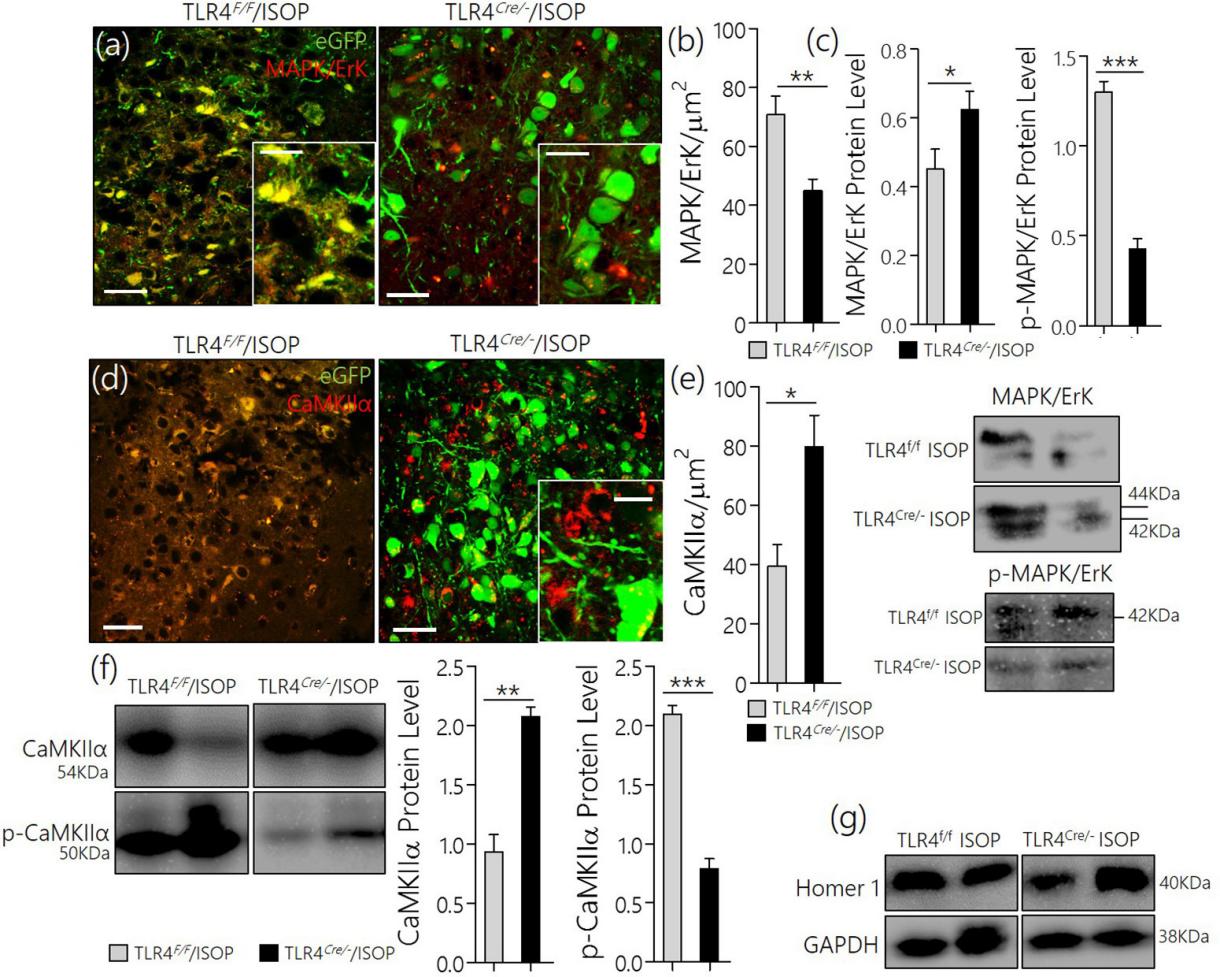
**(a)** Confocal images demonstrating the distribution of MAPK/ErK in TLR4^*loxp*/*loxp*^ and TLR^*Cre*/−^ PVN after ISOP treatment (scale bar = 20 μm). MAPK/ErK was significantly upregulated in the PVN of TLR4^*loxp*/*loxp*^ ISOP group when compared with the TLR4^*Cre*/−^ (^**^p < 0.01). **(b)** Quantitative statistical representation of MAPK/ErK expression in eGFP positive PVN neurons after ISOP treatment. **(c)** Immunoblots showing an increase in total MAPK for the TLR4^*Cre*/−^ mice (^*^p < 0.05) when compared with the TLR4^*loxp*/*loxp*^ PVN. However, a prominent decrease in p-MAPK/ErK was found for the TLR4^*Cre*/−^ mice when compared with the TLR4^*loxp/loxp*^ PVN (^***^p < 0.001). Ultimately, TLR4 knockdown reduced the expression of phosphorylated MAPK. **(d)** TLR4 knockdown (TLR4^*Cre*/−^) in the PVN attenuates CaMKIIa depletion (phosphorylation) after an acute systemic ISOP treatment; when compared with TLR4^*loxp*/*loxp*^ (^*^p < 0.05; scale bar = 20 μm). **(e)** Statistical representation of CaMKIIa expression in the PVN after 48h of ISOP treatment. **(f)** Western blots showing an increase in total CaMKIIa for the TLR^*Cre*/−^ mice (^**^p < 0.01) when compared with the TLR4^*loxp*/*loxp*^ PVN. Furthermore, there was a significant decrease in CaMKIIa phosphorylation (p-CaMKIIa) in the TLR4^*Cre*/−^ group after ISOP treatment; vs. TLR4^*loxp*/*loxp*^ ISOP (^***^p < 0.001). **(g)** Western blots showing the quantitative expression of control proteins Homer-1 and GAPDH for the PVN of TLR4^*loxp*/*loxp*^ and TLR4^*Cre*/−^ PVN groups.

After knockdown of TLR4, TLR4^*loxp*/*loxp*^, and TLR4^*Cre*/−^ (PVN specific knockdown) mice were treated with ISOP (48 h) following which we evaluated the expression of MAPK/ErK and other associated molecules in the PVN; as described previously in WT. A significant decrease in MAPK/ErK expression was observed in the PVN of TLR4^*Cre*/−^ mice, after βARA, when compared with the TLR4^*loxp*/*loxp*^ mice (*p* < 0.05) in confocal microscopy (Figures 6a,b). In subsequent analysis, using western blotting technique, we showed a significant decrease in p-MAPK/ErK (*p* < 0.001), but not MAPK/ErK (*p* < 0.05) in the PVN of TLR4^*Cre*/−^ ISOP group; when compared with the TLR4^*loxp*/*loxp*^ PVN (Figure 6c). The significance of p-MAPK/ErK downregulation in TLR4^*Cre*/−^ PVN was further verified by quantitative analysis of CaMKIIα in eGFP expressing neurons in TLR4^*loxp*/*loxp*^ and TLR4^*Cre*/−^PVN. Interestingly, the loss of CaMKIIα was significantly attenuated in eGFP positive neurons of TLR4^*Cre*/−^ and not in TLR4^*loxp*/*loxp*^ PVN after ISOP treatment (*p* < 0.05; Figures 6d,e). In protein quantification, CaMKIIα expression was significantly higher in TLR4^*Cre*/−^ PVN after ISOP treatment; vs. TLR4^*loxp*/*loxp*^ PVN (*p* < 0.01). Evidently, the TLR4^*loxp*/*loxp*^ ISOP group recorded a significant increase p-CaMKIIα when compared with TLR4^*Cre*/−^ PVN; which showed less p-CaMKIIα (*p* < 0.001; Figures 6f,g).

### TLR4*^*Cre*/−^* rescued inhibitory GABA neurons and reduced VGLUT2 in systemic βARA

**Figure 7:**
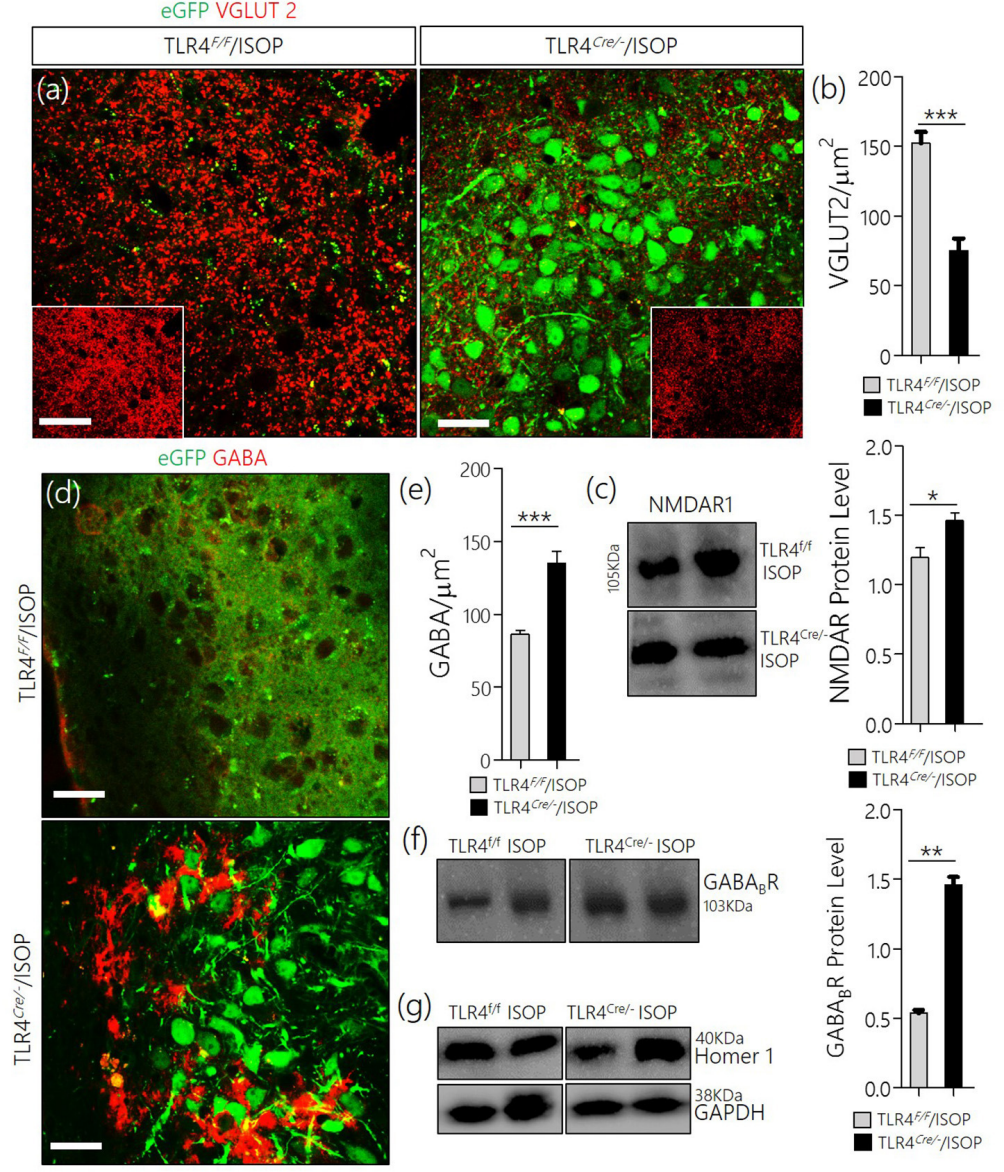
**(a)** Co-localization of eGFP and VGLUT 2 in PVN neurons (scale bar = 20 μm). After ISOP treatment, VGLUT 2 increased profoundly in the PVN of TLR4^*loxp*/*loxp*^ mice when compared with TLR4^*Cre*/−^ (^**^p < 0.001; inset = 40 μm). **(b)** Bar chart representation of VGLUT 2 expression in control and TLR4 knockout PVN. **(c)** Immunoblots showing an increase in NMDAR expression in the PVN of TLR4^*Cre*/−^ mice when compared with TLR4^*loxp*/*loxp*^ mice (^*^p < 0.05). **(d)** βARA caused a decrease in the distribution of GABA in the PVN of TLR4^*loxp*/*loxp*^ mice; vs. TLR4^*Cre*/−^ (^***^p < 0.001; scale bar = 20 μm). **(e)** Statistical representation of GABA expression in the PVN of TLR4^*Cre*/−^ and TLR4^*loxp*/*loxp*^ mice after ISOP treatment. **(f)** Protein quantification analysis show an increase in GABA_B_R expression for the TLR4^*Cre*/−^ ISOP group when compared with TLR4^*loxp*/*loxp*^ ISOP PVN after ISOP treatment (^**^p < 0.01). **(g)** Immunoblots showing the quantitative expression of control proteins Homer-1 and GAPDH for the PVN of TLR4^*loxp*/*loxp*^ and TLR4^*Cre*/−^ PVN groups.

In keeping with the role of TLR4 knockout in the attenuation of MAPK/ErK/CaMKIIα imbalance, there was a corresponding reduction of VGLUT2 (Figures 7a,b), and an upregulation of NMDAR in the TLR4^*Cre*/−^ ISOP when compared with the TLR4^*loxp*/*loxp*^ PVN (Figure 7c). Additionally, our results showed that a change in PVN MAPK/ErK/CaMKIIα expression in TLR4^*loxp*/*loxp*^ ISOP group was associated with a decrease in GABA (*p* < 0.001; Figures 7d,e) and GABA_B_R (*p* < 0.01; Figures 7f,g); like the WT/ISOP. However, in the TLR4^*Cre*/−^ PVN, after ISOP treatment, GABA and GABA_B_R depletion was rescued; vs. the TLR4^*loxp*/*loxp*^ PVN (Figures 7d,g).

### TLR4*^*Cre*/−^* reduced sympathoexcitation in βAR activation

**Figure 8:**
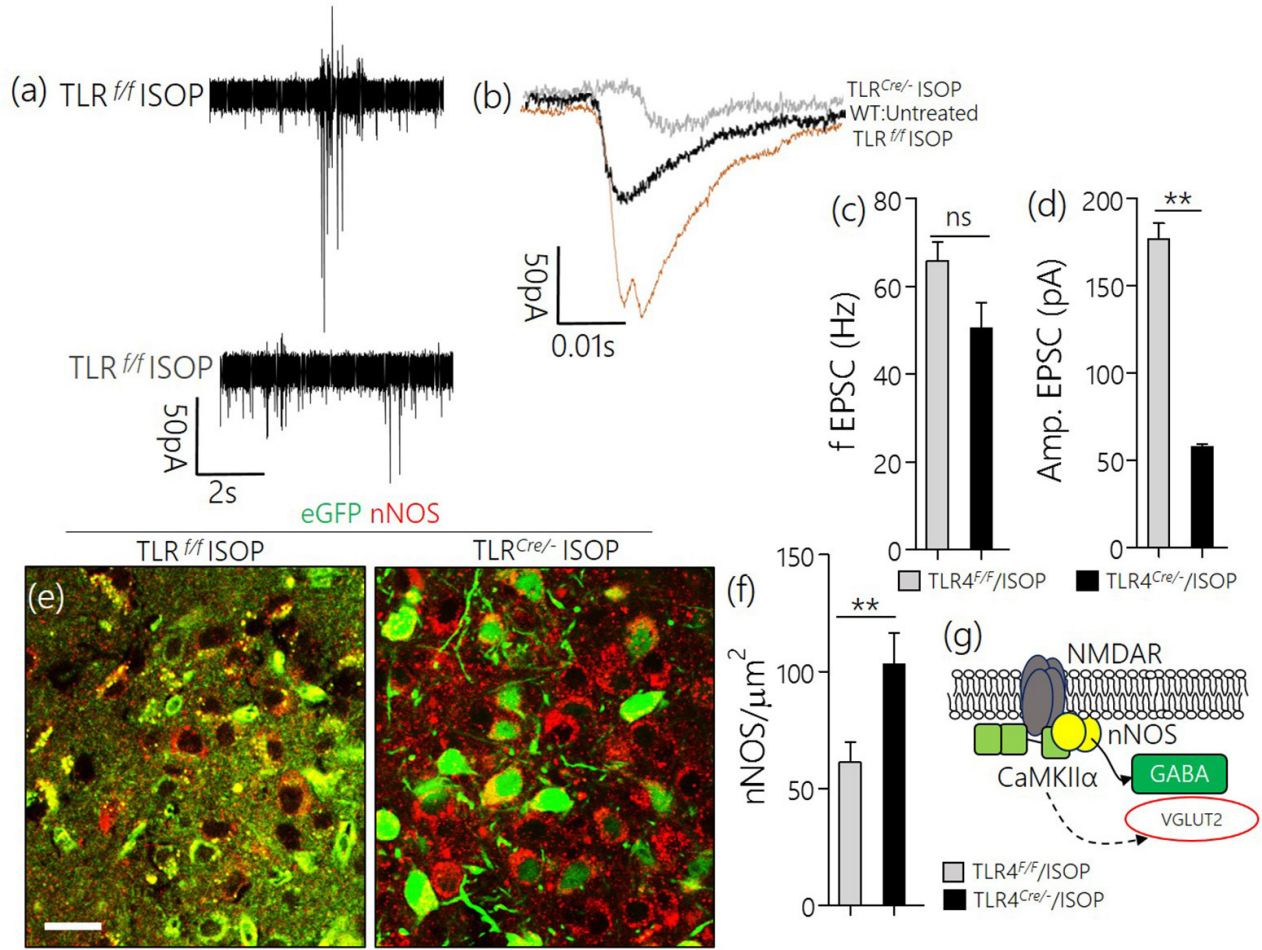
**(a,b)** Spontaneous EPSC recording from the PVN of ISOP treatment (in vivo) mice. While the frequency of EPSCs increased for both TLR4^*Cre*/−^ and TLR4^*loxp*/*loxp*^ PVN neurons (ns), the amplitude decreased significantly in the TLR4 knockout group (^**^p < 0.01). **(c,d)** Bar chart illustrating a change in frequency and amplitude of EPSCs for ISOP treated TLR4^*Cre*/−^ and TLR4^*loxp*/*loxp*^ mice. **(e)** Confocal images showing the colocalization of eGFP and nNOS in the PVN. The distribution of the neuronal stress marker reduced significantly in the TLR4^*loxp*/*loxp*^ mice when compared with the TLR4^*Cre*/−^ groups after ISOP treatment (scale bar = 20 μm). **(f)** Bar chart showing the relative change in PVN nNOS expression for the TLR4^*loxp*/*loxp*^ and TLR4^*Cre*/−^ mice after systemic ISOP treatment. **(g)** Schematic illustration of the mechanism through which synaptic expression of nNOS can depict a change in the activity of GABA or Glutamate in the PVN.

Similar to the observation in WT/ISOP PVN, TLR4^*loxp*/*loxp*^ ISOP PVN neurons showed an increase in the frequency and amplitude of spontaneous EPSCs when compared with the WT control (Figures 8a,b). Interestingly, after TLR4 knockout, there was an increase in frequency but not the amplitude of EPSCs in the PVN neuron (*p* < 0.01, ns; Figures 8c,d). As a confirmation of a reduction in sympathoexcitation see Sharma et al. (2011), we found that the expression of nNOS was significantly higher for the TLR4 ^*Cre*/−^ ISOP; when compared with the TLR4^*loxp*/*loxp*^ ISOP group (Figure 8e–g; *p* < 0.01).

### Reduced sympathoexcitation and cardiac tissue morphology

**Figure 9:**
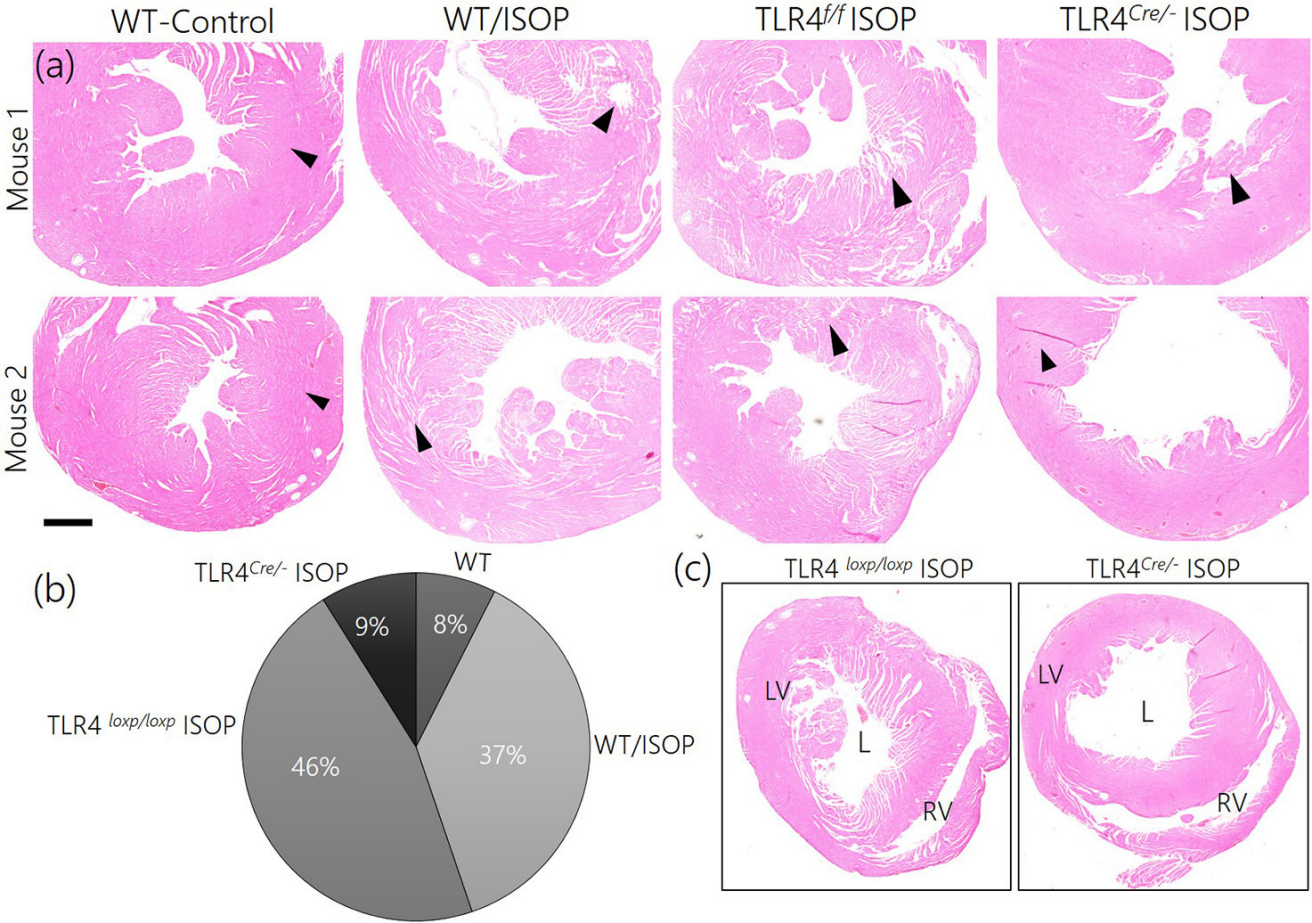
**(a)** ISOP administration resulted in a change in cardiac tissue morphology for the ISOP treatment groups (WT/ISOP and TLR4^*loxp*/*loxp*^ ISOP) when compared with the control. For the TLR4 knockdown group (TLR4^*Cre*/−^ ISOP), there was no observable damage to the ventricular wall when compared with the control. **(b)** Descriptive pie chart showing a normalized percentage area affected by systemic ISOP treatment. The widest affected areas were recorded in WT/ISOP and TLR4^*loxp*/*loxp*^ ISOP groups. Arrow heads indicate the wall of the left ventricle. **(c)** Low magnification comparison of right and left ventricular morphology after ISOP treatment in TLR4^*loxp*/*loxp*^ and TLR4^*Cre*/−^ group. The TLR4^*loxp*/*loxp*^ showed a significant change in ventricular wall when compared with the TLR4^*Cre*/−^ group.

Since TLR4 knockout in the PVN reduced the imbalance between MAPK/ErK and CaMKIIα, we sought to elucidate if this contributes to the overall profile of cardiac tissue morphology (histology). After 48 h of ISOP treatment, we confirmed sympathoexcitation by serologically determining the areas affected systemic βARA in ventricular wall (Figures 9a,b). While WT/ISOP and TLR4^*loxp*/*loxp*^ ISOP showed significant changes in ventricular wall, no prominent change was recorded in the TLR4^*Cre*/−^ ISOP (Figure 9c). However, there was an increase in lumen size for the TLR4^*Cre*/−^ ISOP group when compared with the control.

In support of this outcome, we observed increase in cardiac thickness after 48 h of isoproterenol treatment in WT mice as reflected by a significant increase in the thickening of interventricular septum in systole, posterior wall thickness in both systole and diastole. In addition, WT mice infused with ISO showed an increase in heart rate. In contrast, treatment of TLR4 knockout mice with ISO did not increase ventricular thickness or heart rate. In agreement with echocardiographic finding, histological finding revealed thickening of the wall without an increase in LV cavity size indicating enhanced contraction in WT mice treated with ISO but not in the TLR4 knockout mice. These finding indicate beta adrenergic stimulation increased cardiac contractility and this effect was blunted in TLR4 knockout mice indicating a role for inflammatory molecule in cardiac dysfunction and exaggerated sympathetic activity in mice (Table 1).

**Table 1:** Values are means ± SE.

**Parameters**	**Exp. groups**	**Baseline**	**48 h**
IVSd (mm)	WT+ISO	0.70 ± 0.02	0.77 ± 0.01
	TLR4+ISO	0.74 ± 0.01	0.76 ± 0.01
IVSs (mm)	WT+ISO	1.03 ± 0.02	1.22 ± 0.02^*^
	TLR4+ISO	1.04 ± 0.01^#^	0.97 ± 0.01^$^
LVIDd (mm)	WT+ISO	3.70 ± 0.02	3.77 ± 0.03
	TLR4+ISO	3.74 ± 0.03	3.84 ± 0.03
LVIDs (mm)	WT+ISO	2.32 ± 0.01	2.30 ± 0.02
	TLR4+ISO	2.34 ± 0.01	2.33 ± 0.01
LVPWd (mm)	WT+ISO	0.77 ± 0.01	0.87 ± 0.01
	TLR4+ISO	0.74 ± 0.01	0.78 ± 0.01^$^
LVPWs (mm)	WT+ISO	1.15 ± 0.03	1.37 ± 0.02^*^
	TLR4+ISO	1.08 ± 0.03^#^	1.13 ± 0.01^$^
FS (%)	WT+ISO	35.68 ± 0.71	37.52 ± 0.33
	TLR4+ISO	35.68 ± 0.45	36.14 ± 0.24
HR	WT+ISO	479.0 ± 4.81	584.79 ± 7.98^*^
	TLR4+ISO	487.4 ± 6.85^#&^	510.20 ± 5.35^@^

*IVSd and IVDs, interventricular septal thickness in diastole and systole, respectively; LVIDd and LVIDs indicate left ventricular internal diameter at diastole and systole, respectively; LVPWd and LVPWd, left ventricular posterior wall thickness at diastole and systole, respectively; FS, fractional shortening and HR, heart rate*.

p < 0.05-WT − Baseline vs. WT + ISO 48 h^*^, p < 0.05-WT + ISO 48 h^$^ vs. TLR4KO - Baseline^#^, p < 0.05-WT + ISO 48 h vs. TLR4KO 48 h^$^, p < 0.05-WT Baseline vs. TLR4KO 48 h^&^, p < 0.05-WT + ISO 48 h vs. TLR4KO Baseline^#^, and p < 0.05-WT + ISO 48 h vs. TLR4KO 48h^@^.

*Data were submitted to multivariate analysis of variance (MANOVA) and then compared between groups and moments by Tukey-Cramer*.

## Discussion

The outcomes of this study suggest that systemically driven βARA causes a change in phosphorylation and expression of synaptic kinases (MAPK/ErK/CaMKIIα) in the PVN. This was generally associated with a significant increase in p-MAPK/ErK, and phosphorylation of synaptic regulatory kinase (CaMKIIα). Furthermore, there was a change in the distribution of transporter and receptor components of Glutamate/GABA neurotransmission within the PVN after an acute—*in vivo*—systemic ISOP treatment. Taken together, we showed that pertaining to an acute systemic βARA, an upregulation of PVN p-CaMKIIα, and associated neurochemical changes were mediated—in part—through the neural MAPK/TLR4 axis. As such, a change in PVN MAPK/ErK/CaMKIIα phosphorylation pattern may be central to the synaptic changes observed in response to systemic βARA.

MAPK/ErK signaling is an integral part of TLR4 activation associated with neuroinflammatory responses in the CNS (Badshah et al., 2016; Cho et al., 2016). Furthermore, MAPK/ErK activation, downstream of TLR4, may contribute to the phosphorylation (inactivation) of CaMKIIα at post-synaptic sites (Liu et al., 2010; Li et al., 2015). Recent studies have described the role of neural inflammation in systemic cardiovascular changes. Moreover, there is substantive evidence to suggest that deletion of cardiac TLR4 is protective against systemic ISOP-induced necrosis (Kim et al., 2006). Yet, it has been unclear whether a systemic driven change in cardiovascular function can directly alter PVN synaptic function.

The PVN has initially been described as a mere relay station for neurons of the descending hypothalamic-hypophysial systems (Babović et al., 2009). However, morphological and electrophysiological characterization of PVN neurons shows that it is made up of several integrated excitatory and inhibitory neural circuits concerned with the regulation of autonomic cardiovascular functions (Pyner, 2009; Glass et al., 2015; Pandit et al., 2015; Ogundele et al., 2016a). An important component of the pre-autonomic sympathetic control is the activity of adrenergic neurotransmitters in parts of the brain connected to the PVN (Jung et al., 2004). Specifically, the rostral ventrolateral medulla (RVLM), NTS, and catecholaminergic neuron group (C1) of the brain stem receives autonomic inputs from the PVN and projects to the intermediolateral column of the spinal cord (thoracolumbar region). This then innervates the heart through the cardiac plexus (Menani et al., 2014; Van Kempen et al., 2015; Ogundele et al., 2016b).

Physiologically, the state of the excitatory and inhibitory neurotransmitters, within the PVN, is an indirect representation of the magnitude of sympathoexcitation (Zhang et al., 2002; Li and Pan, 2007). While a systemic βAR activation may increase the heart rate by stimulating β2 receptors in the heart (Yusuf et al., 1987), the outcome of this study suggests that such an activation may alter pre-autonomic events. As such, since ISOP does not readily cross the blood-brain barrier (Olesen et al., 1978), it is logical to assume that the changes observed here were to a large extent part of a wide and diffuse response to cardiac mechanical receptor activation, rather than β2R signaling within the PVN. Interestingly, this effect may occur in reverse. As such, neural factors which promotes excitatory activity, or decrease inhibitory neurotransmission in the PVN has been shown to increase sympathoexcitation (Patel et al., 2016).

Substantive evidence now exists to suggest that β_2_R activation generates a significant level of MAPK/ErK in the tissue through the cyclic adenosine monophosphate/protein kinase A signaling pathways (Zheng et al., 2000). Other studies have shown a direct relationship between the release of calcium and MAPK generated after β_2_R activation in cardiomyocytes (Magne et al., 2001). Aside from their role in the regulation of synaptic dysfunction and calcium release, the MAPK and CaMKIIα are also involved inflammatory signaling downstream of *TLR4* (Wang et al., 2014; Swaroop et al., 2016). Since MAPK/ErK is a major signaling molecule acting downstream of TLR4, we asked whether targeting PVN TLR4 may attenuate some of the neurochemical changes associated with systemic βAR activation-mediated MAPK/CaMKIIα imbalance. Like the observation in cardiac tissue, the outcome of this study showed that TLR4 knockout attenuates PVN MAPK/ErK phosphorylation (Figures 6a–c) after an acute systemic βAR activation when compared with the WT. Furthermore, this prevented loss of CaMKIIα, and restored excitatory/inhibitory neurotransmitter balance (Figures 6d–f). The significance of a change in MAPK/ErK expression in eGFP positive neurons for TLR4^*Cre*/−^ISOP (Figure 6a) vs. its expression in PVN total lysate (Figure 6b) cannot be over emphasized. While we have highlighted the expression of MAPK/ErK co-localized with eGFP in microscopy, these images did not highlight possible changes in astroglia MAPK/ErK or p-MAPK/ErK. However, in immunoblots, all sources of MAPK/ErK and p-MAPK/ErK were considered.

To determine the relationship between CaMKIIα and TLR4 expression patterns, we quantified total MAPK/ErK and, phosphorylated MAPK/ErK in the PVN of ISOP treated mice. Although there was an increase in total MAPK/ErK in PVN total lysate (Figure 6b; *p* < 0.05), confocal imaging revealed a decrease in MAPK/ErK—specifically—for eGFP positive PVN neurons in TLR4^Cre/−^ISOP group (Figure 6a; *p* < 0.01). Since it has been established that p-MAPK/ErK phosphorylates CaMKIIα (Mizukami et al., 2015; Yu et al., 2016), we further quantified the expression of PVN p-MAPK/ErK in the PVN. Although TLR4^*Cre*/−^ISOP group recorded a higher PVN MAPK/ErK expression, there was a significant decrease in p-MAPK/ErK when compared with the TLR4^*loxp*/*loxp*^ ISOP group (*p* < 0.001) after ISOP treatment. In subsequent analysis, we deduced that these outcomes may hold a direct effect of CaMKIIα expression and phosphorylation pattern. As such, a decrease in p-MAPK/ErK in the PVN of TLR4^*Cre*/−^ISOP group was associated with an increased expression of CaMKIIα (*p* < 0.01), but not p-CaMKIIα when compared to the TLR4^*loxp*/*loxp*^ ISOP group—which showed the opposite (Figures 6d–f).

### TLR4 knockout attenuated sympathoexcitation in βARA

Here we showed that an acute βAR activation was associated with alterations in preautonomic synaptic substrates. This was generally accounted for a change in glutamatergic and GABAergic systems (Figure 2). Although VGLUT2 expression increased in the preautonomic area, there was a significant decrease in NMDAR expression in the PVN of ISOP treated mice. However, a striking decrease in VGAT/GABA and GABA_B_R suggests a decreased inhibition of the PVN during an acute systemic ISOP treatment. In support of this proposition, a decrease in GABA and GABA_B_R was associated with an increased threshold and frequency for spontaneous EPSCs in the preautonomic area (Figure 4).

TLR4 may alter the expression of nNOS through the activity of MAPK/ErK at synaptic densities (Yao et al., 2012; Wang et al., 2016). Moreover, a reduction in the expression of nNOS, during inflammation, has been described in the loss of NO and GABA activity (Lethbridge et al., 2012; Di Mauro et al., 2013). Ultimately, our results showed that an increase in sympathoexcitation may involve MAPK-mediated glutamatergic activity or nNOS-related GABA loss after βARA. While nNOS is required for NO-mediated GABAergic activation, a reduction in the tissue level of NO and GABA has been described in the PVN after a phase of TLR4-mediated inflammation (Affleck et al., 2012). Furthermore, the activity of downstream pro-inflammatory molecules- such as NF-κB and IL-1β—have been identified in nNOS/GABA inhibition in the PVN (Crowley et al., 2015; Dange et al., 2015). Based on our results, and reports by other groups (Kim et al., 2006; Dange et al., 2015; Crowley et al., 2015), we deduced that βARA caused sympathoexcitation, in part, through TLR4/MAPK/ErK-mediated loss of GABA activity in the PVN. As such, when a tissue—specific TLR4 knockdown was implemented, we observed no significant loss in GABA and nNOS expression in the PVN (TLR4^*Cre*/−^) after ISOP treatment. TLR4 knockdown also had a significant effect on the amplitude, but not the frequency of EPSCs in the PVN. As such, there was no significant change in fEPSC, while the TLR^*Cr*/*e*−^ ISOP responses exhibited a lower threshold vs. TLR4^*loxp*/*loxp*^ ISOP.

It is noteworthy to mention that methods such as RT-PCR can serve as useful tools in adequately measuring the extent of knockdown attained using the AAV cocktail and protein expression. However, western blotting gave us an overall outcome on protein expression pattern. This was ultimately used to determine the extent of the knockdown in PVN lysate. While our study has opened a new premise for assessing the crosstalk between PVN synaptic kinases and systemic cardiovascular activation, there are several gaps that our study does not address. Majorly, since ISOP remains systemic thereafter, the actual mechanism through which these changes may occur remains open for further investigation. A logical explanation may involve a broad up-regulation of p-MAPK (systemic) capable of causing changes in blood vessels, and permeability of the blood brain barrier (González-Mariscal et al., 2008; Pan et al., 2011). Additionally, systemic ISOP treatment may involve neural stress response mechanism—through adrenergic systems—and oxidative stress (Rathore et al., 2000) that is capable of eliciting changes in the PVN. Therefore, there is a need for further characterization of stress response systems, and determining the profile of the blood-brain barrier after acute ISOP treatment.

## Summary

Although systemic ISOP does not effectively cross the blood–brain barrier, we found that acute systemic βAR activation may indirectly affect the neurochemical balance of the pre-autonomic PVN through changes in CaMKIIα/MAPK/ErK, downstream of TLR4. Consequently, truncating the TLR4 pathway attenuated CaMKIIα loss, and restored excitatory/inhibitory neurotransmitter balance to certain extent.

## Author contributions

OO, FR, and RD: Conducted aspects of the experiment, analysis and manuscript write up. CL: Supervised the research, conducted aspects of the experiments and revised the manuscript. JF, CL, and RD: Supervised aspect of the research design and implementation, contributed in analysis and edited the final write up.

### Conflict of interest statement

The authors declare that the research was conducted in the absence of any commercial or financial relationships that could be construed as a potential conflict of interest.
