# Supplementary figures and images for: Systemic Sympathoexcitation Was Associated with Paraventricular Hypothalamic Phosphorylation of Synaptic CaMKIIα and MAPK/ErK
Source: Front Neurosci. 2017 Aug 3;11:447. doi: 10.3389/fnins.2017.00447 (PMC5541931; doi:10.3389/fnins.2017.00447)

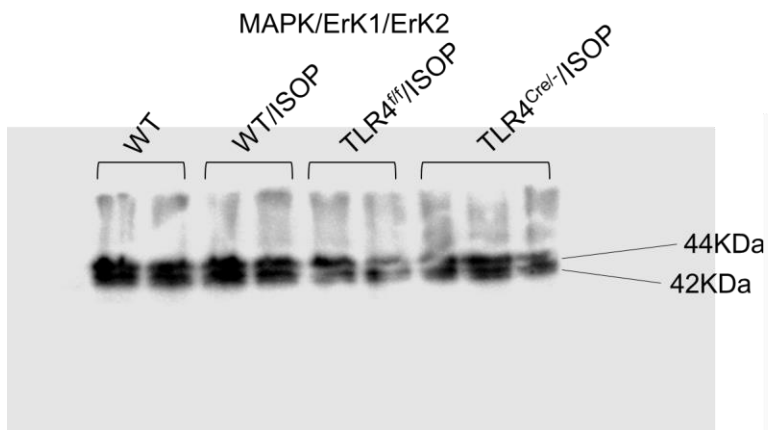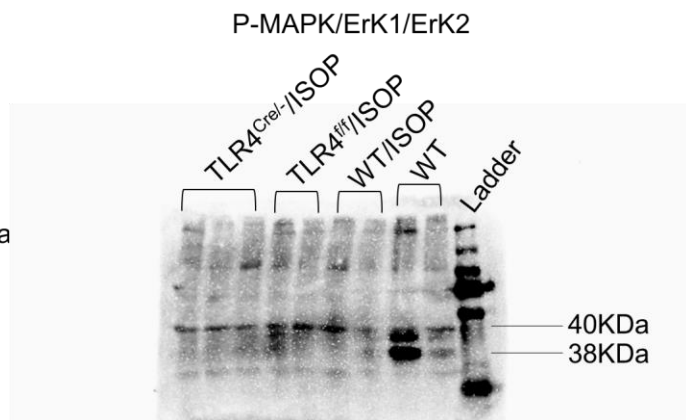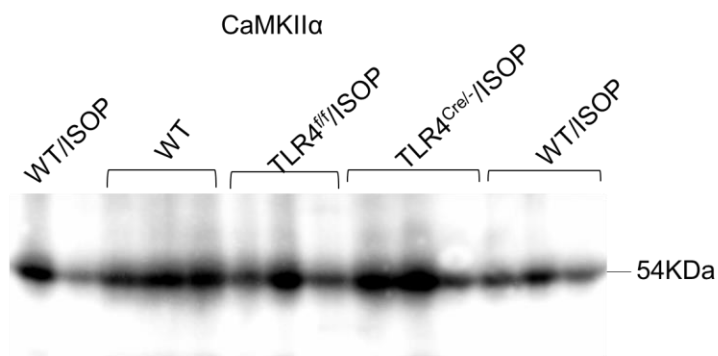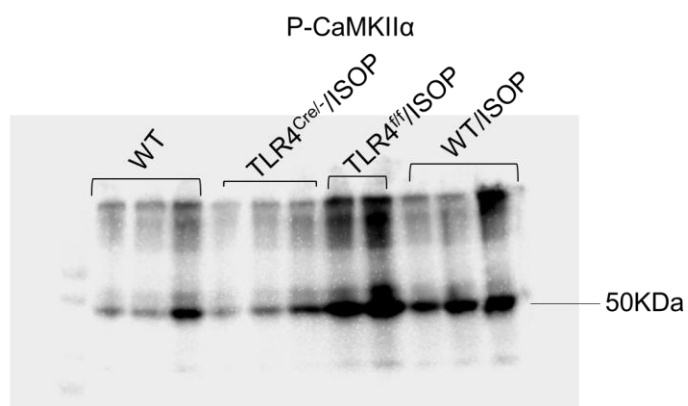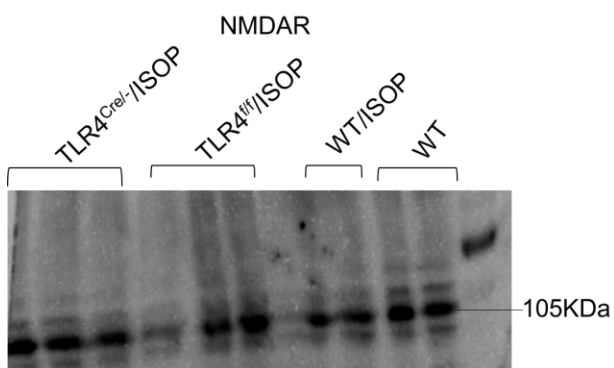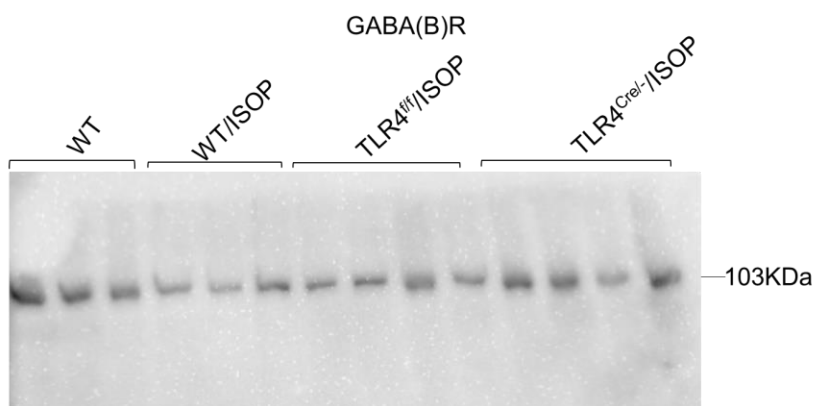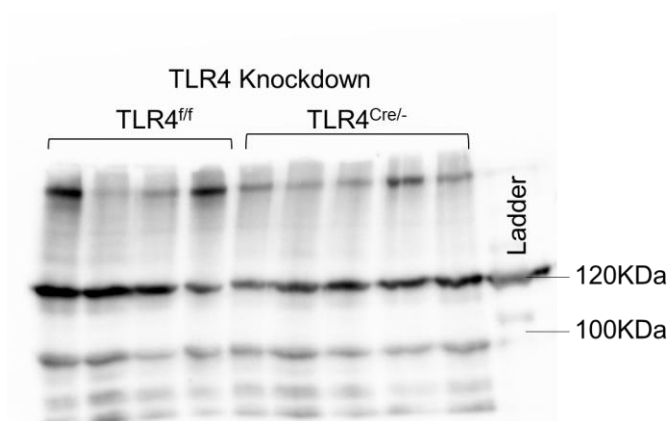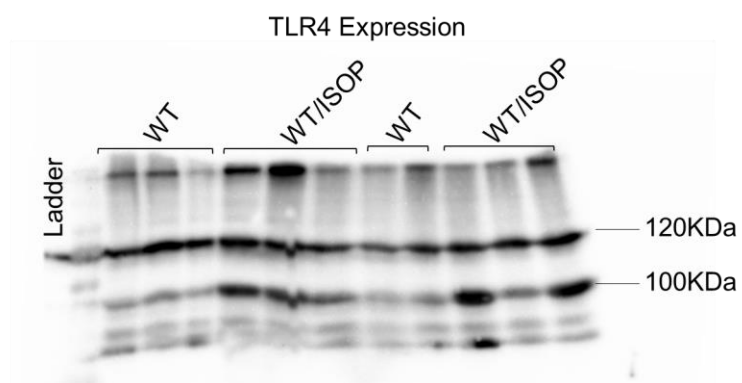

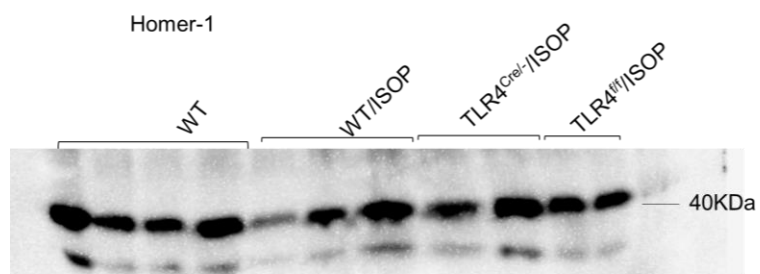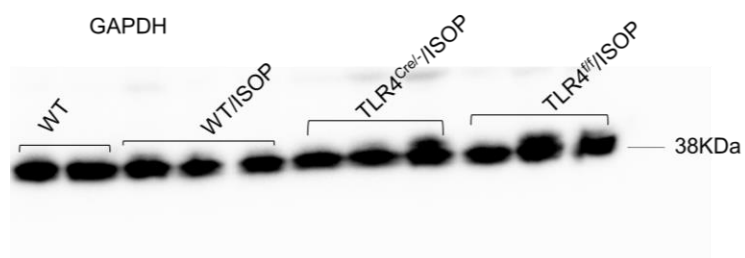

Supplement: Supplementary file 1 [file DataSheet1.PDF]
